# Supplementary figures and images for: Differentially expressed tRNA-derived fragments in bovine fetuses with assisted reproduction induced congenital overgrowth syndrome
Source: Front Genet. 2022 Nov 15;13:1055343. doi: 10.3389/fgene.2022.1055343 (PMC9705782; doi:10.3389/fgene.2022.1055343)

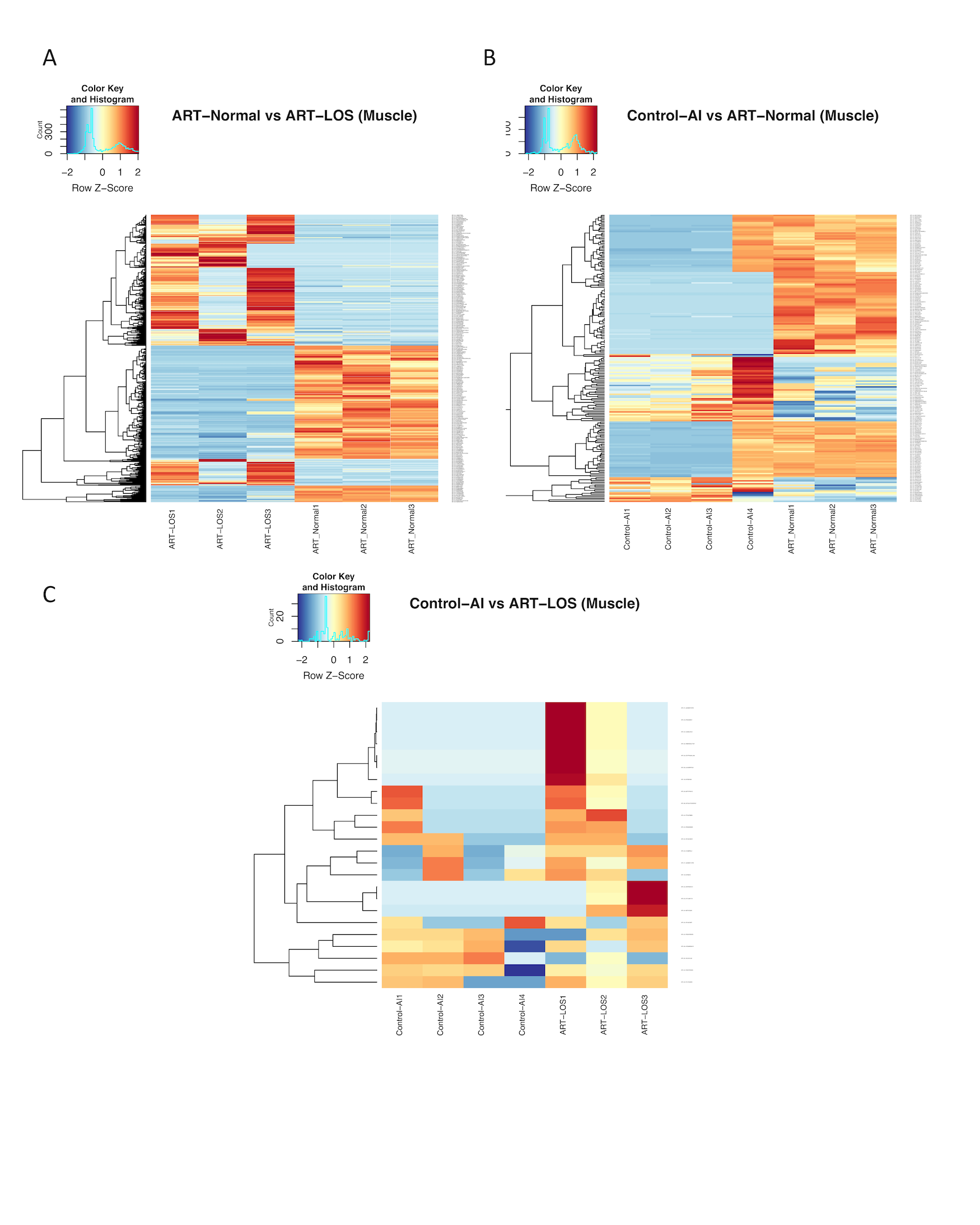

Supplement: Supplementary file 1 [file Image3.TIFF]

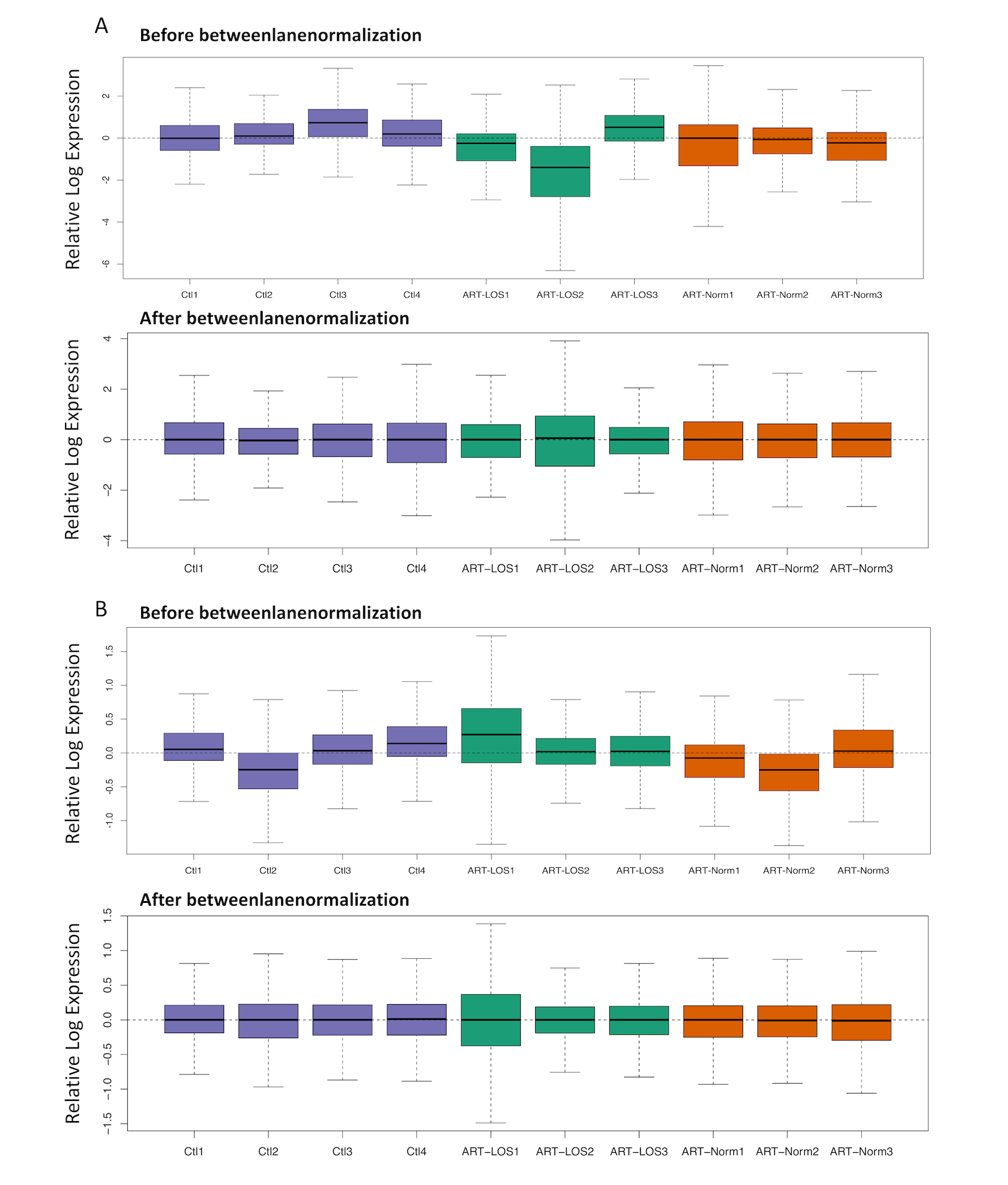

Supplement: Supplementary file 3 [file Image1.TIFF]

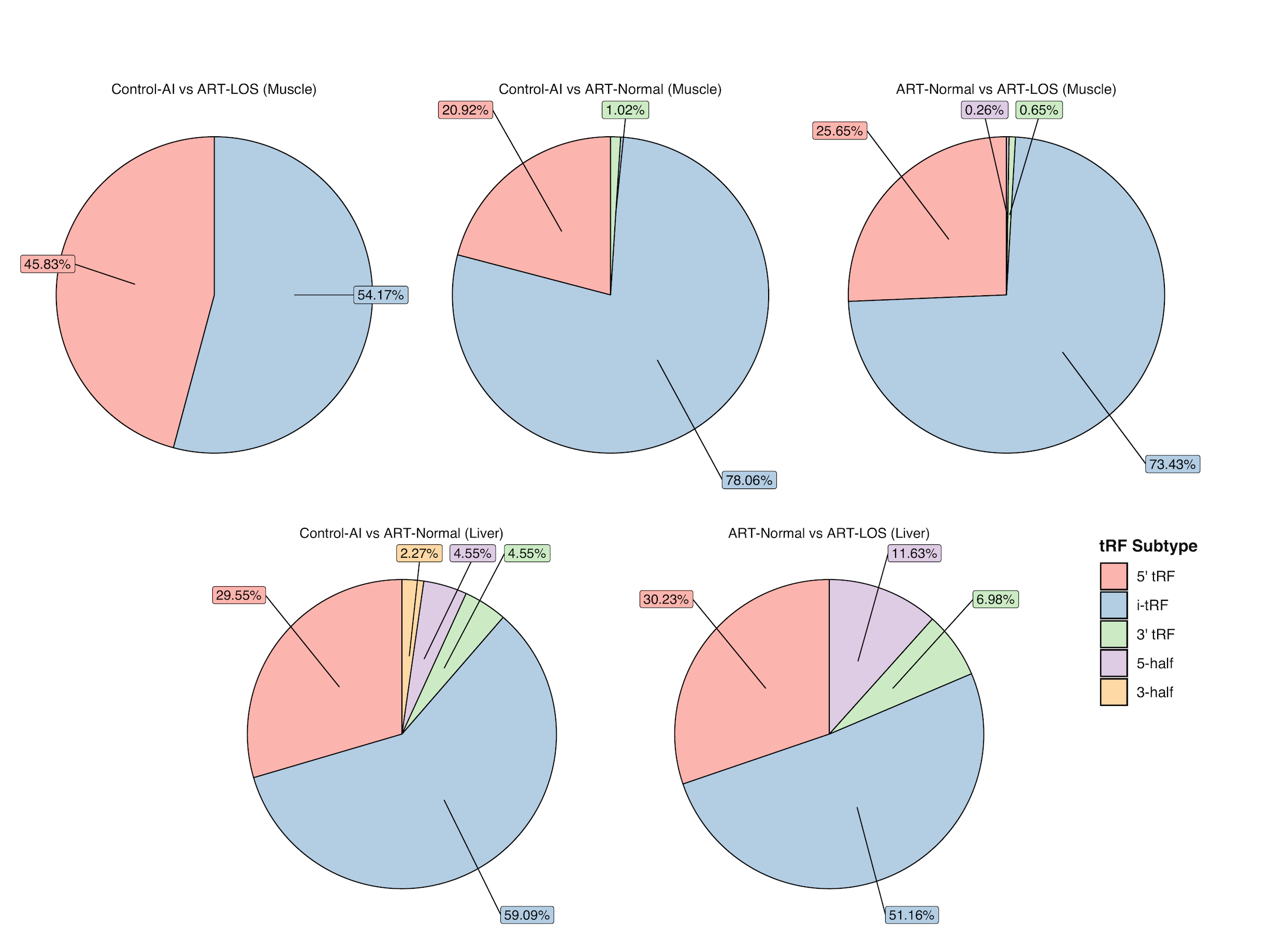

Supplement: Supplementary file 5 [file Image5.TIFF]

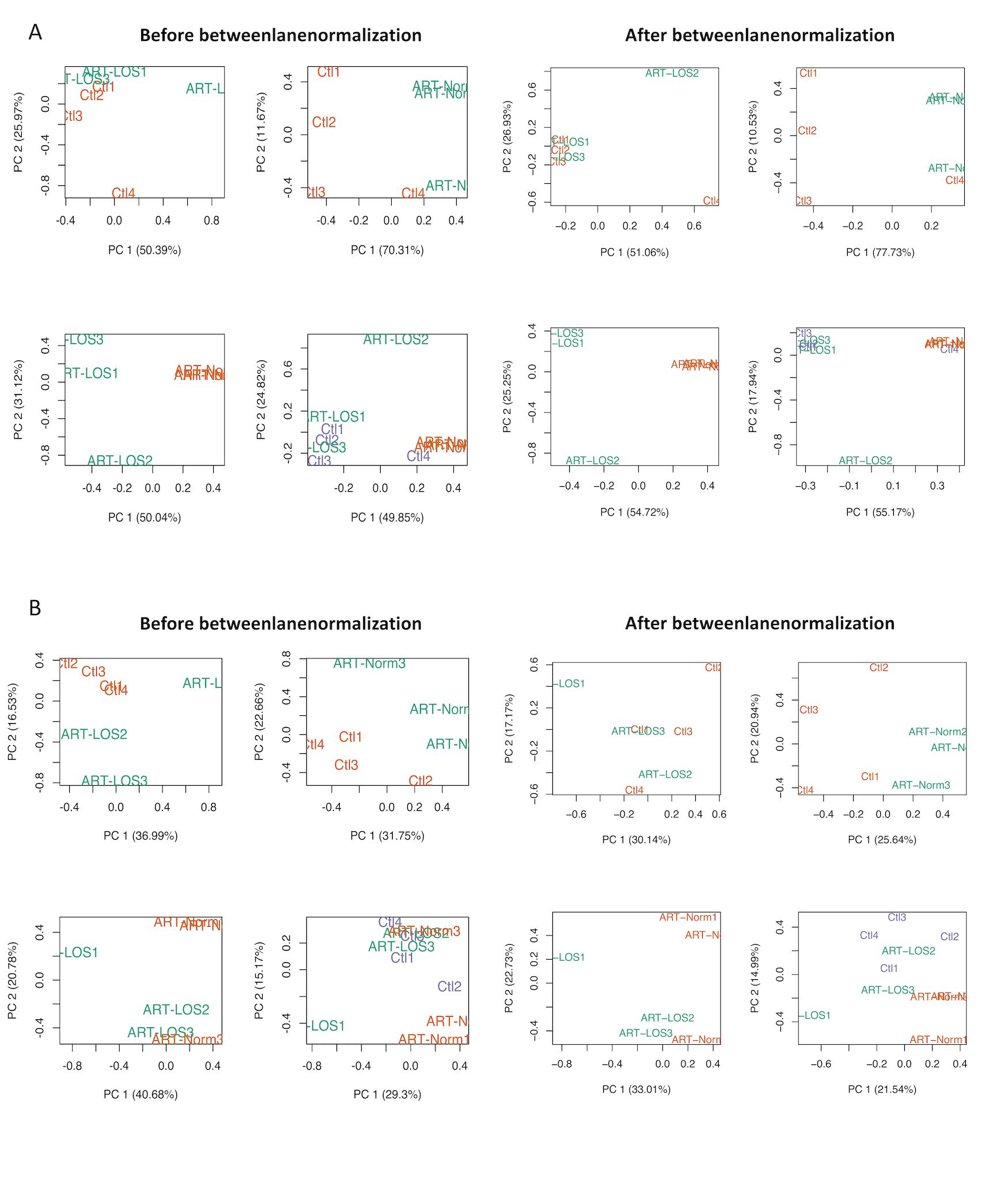

Supplement: Supplementary file 8 [file Image2.TIFF]

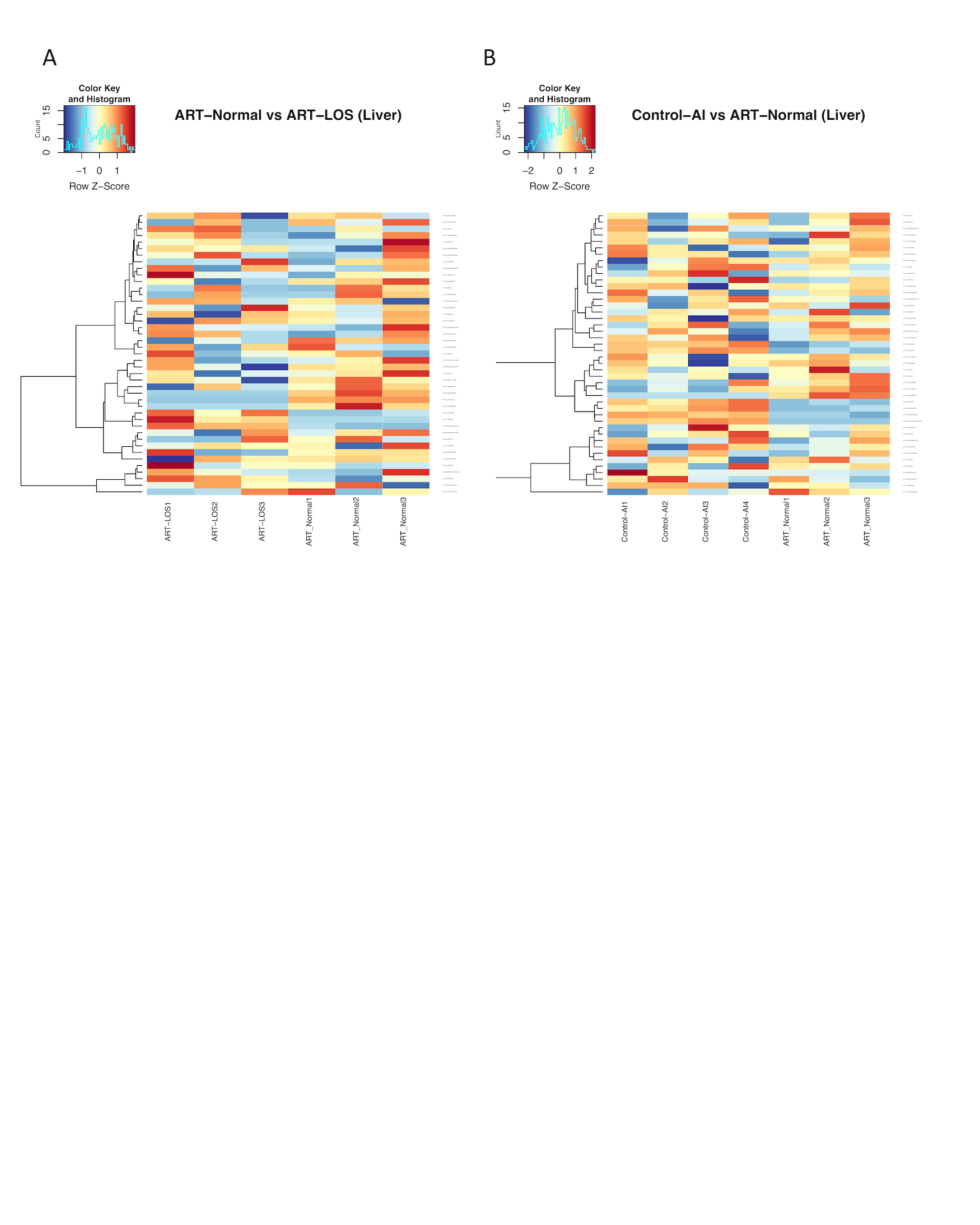

Supplement: Supplementary file 9 [file Image4.TIFF]
